# *In Vitro* Characterisation of 3D Printable Filaments Subjected to Edible Liquids: An Analysis of Fused Deposition Modelling for Intraoral Applicability

**DOI:** 10.1155/2024/2118412

**Published:** 2024-08-30

**Authors:** Nafij Bin Jamayet, Aparna Barman, Farah Rashid, Sumaiya Zabin Eusufzai, Mutlu Özcan, James Dudley, Taseef Hasan Farook

**Affiliations:** ^1^Division of Restorative Dentistry, School of Dentistry, IMU University, Kuala lumpur 57000, Malaysia; ^2^School of Dental Sciences, Universiti Sains Malaysia, Kota Bharu, Malaysia; ^3^Adelaide Dental School, The University of Adelaide, Adelaide, South Australia, Australia; ^4^Center of Dental Medicine, University of Zurich, Zurich 8032, Switzerland

## Abstract

**Purpose:**

To evaluate the influence of edible liquids on the characteristic properties of 3D printable materials compared to conventionally used dental resin acrylic.

**Method:**

Dental polymethyl methacrylate (PMMA) specimens were fabricated from preformed molds while polylactic acid (PLA) and polyethylene terephthalate glycol (PETG) specimens were 3D printed using fused deposition modelling at 0.1 mm layer thickness. All specimen forms adhered to ISO 37:2017 and ISO 604:2002 specifications. Specimens underwent controlled immersion for 180 hr into different media (no immersion (control), oil, soda, milk, and caffeine). Changes in mass (g), plastic deformity (N/mm^2^), ultimate tensile stress (N), flexural deformity (N/mm^2^), and break force (N) were evaluated using analysis of variance.

**Results:**

There was an increase in mass for all specimens following immersion with significant interactions between immersion media and the materials. The materials exhibited significant differences in plastic deformity (*F* (df) = 156.632(2), *P*  < 0.001), ultimate tensile stress (*F* (df) = 109.521(2), *P*  < 0.001), and break force (*F* (df) = 319.785 (2), *P*  < 0.001) with no significant interactions with immersion media (*P* > 0.05) on both accounts. Materials showed no significant differences in flexural deformity (*F* (df) = 2.693(2), *P* = 0.074) but with significant interactions (*F* (df) = 4.984(8), *P*  < 0.001) between acrylic and immersion media.

**Conclusion:**

Commercially available 3D printable PLA and PETG filaments printed at 0.1 mm thickness possess approximately half the mechanical resilience as dental PMMA with comparable flexural deformity after exposure to edible liquids.

## 1. Introduction

Intraoral dental prostheses need to withstand flexural and masticatory tensile forces during eating [1]. Such properties are generally affected by the person's daily dietary habits (i.e., acidic diets) [2]. Conventional intraoral prostheses are made of biocompatible polymethyl methacrylate (PMMA) resin which satisfy critical aesthetic, functional, and mechanical properties [2], but as dentistry is transitioning towards digitisation, additive manufacturing and 3D printing are receiving special attention due to its freedom in digital designs, excellent precision, and less material wastage [3, 4].

Customised digital implants and prosthetics, 3D diagnostic models, occlusal splints, clear aligners, and surgical trays are now being 3D printed using various print materials [5]. Biocompatible and biodegradable polylactic acid (PLA) is used in implants and tissue staging [6, 7], while polyethylene terephthalate glycol (PETG), known for its excellent thermomechanical and thermoplastic properties [8, 9], is used in dental aligners and fixed orthodontic appliances [8]. Both materials are inexpensive and readily available [10]. Each 3D printable material has unique physical characteristics that make them uniquely suited for specific dental procedures [11]. PLA has a low glass transition temperature and melting point, resulting in lower thermal stability and higher susceptibility to degradation from moisture and certain chemicals. However, it is biodegradable and environmentally friendly. PETG, on the other hand, offers superior thermal stability, higher chemical resistance, and greater impact resistance, making it more durable but not biodegradable. PMMA is known for its excellent optical clarity and provides good thermal stability and chemical resistance, though it is more brittle than PETG and not biodegradable [12]. While both PETG and PMMA are recyclable, they are derived from petroleum-based sources, making them less environmentally friendly than PLA. However, the influence of diet on the longevity of such 3D printable materials as intraoral prostheses has not yet been tested [13].

Commercial soybean oil is a composition of various acids (lauric, myristic, palmitic, stearic, oleic, linoleic, etc.) and is mostly used for cooking purposes [14, 15]. Asian cooking is renowned for curries of very high oil content [16]. Nonalcoholic acidic beverages like soda are heavily consumed (50 gallons/person and 34% of world adult population) across the globe and are therefore specially addressed by various WHO dietary directives [17, 18, 19]. Milk and lactose product consumptions have seen a substantial rise since 2003 with an estimated 57% of developing nation populations consuming milk in 2020 [20]. Surveys from the UK and the USA suggest that 48%–90% of the population consume caffeine in some form, usually tea or coffee [21, 22, 23]. 3D printable materials provisioned for intraoral applications should be able to withstand such commonly consumed food substances.

The current *in vitro* study aimed to evaluate the influence of edible liquids, (1) cooking oil, (2) nonalcoholic carbonated beverage, (3) milk, and (4) caffeine, on the physical properties (mass, plastic deformity, ultimate tensile stress, flexural deformity, and break force) of PLA and PETG printable materials. The null hypothesis tested was that there will be no significant difference in the mechanical properties for PLA and PETG when compared to the conventional PMMA after being subjected to common edible liquids.

## 2. Methods

### 2.1. Sample Size Estimation

Based on an effect size of 0.64 [24], with *α* = 0.05 and a power of 0.95, a fixed effects model with interactions for the three materials across five methods of immersion was proposed, resulting in 15 overall groups. This model recommended a sample size of 59 across the three material classes. Sample sizes were estimated using G-Power 3.1.9.4. An additional 30% of samples were considered, based on the highest sample size recommended for each test, to counteract any handling errors. Therefore, 90 samples were fabricated for tensile property testing (tens models), and 90 samples were made for flexural property testing (flex models).

### 2.2. Preparation of the 3D Materials

**Figure 1:**
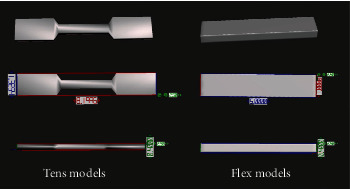
Resultant dimensions (in centimetres) of the tens and flex CAD models.

All models were designed using CAD software (FreeCAD; Jurgen Riegel, Germany) in adherence to the ISO standards mentioned below. The main two forms fabricated were “test models for plastic deformity and ultimate tensile stress (tens models)” and “test models for flexural deformity and break force (flex models)”. Following the finalisation of the design, the dimensions of the models prior to 3D printing are shown in Figure 1. To aid reproducibility, the CAD files and the subsequent 3D printable files are provided as supplementary file folder labelled *Supplementary 1*.

#### 2.2.1. Test Models for Plastic Deformity and Ultimate Tensile Stress (Tens Models)

**Figure 2:**
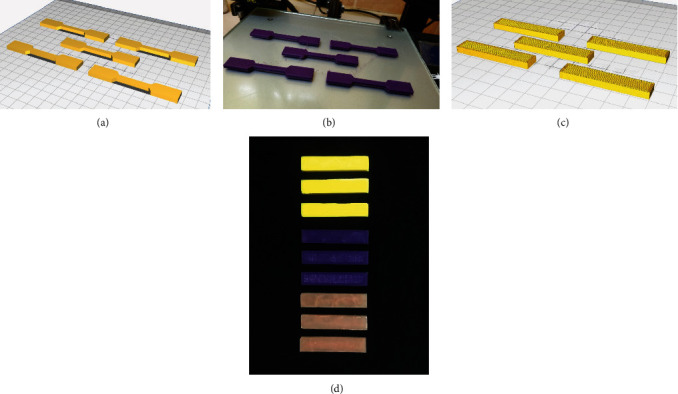
(a) Design of tens models in CAD. (b) 3D printed tens models. (c) Design of flex models in CAD. (d) Printed specimens using coloured filaments to code specimens according to underlying material (yellow = PETG, purple = PLA, and pink = acrylic).

Silicone moulds were prepared to create PMMA specimens (tens models) according to (ISO 37:2017) [25]. Thirty PMMA tens models were made using dental acrylic (Vertex Dental B.V; Netherlands). Excess materials and rough edges were reduced using scissors and sandpaper (P120 Merka; Finland). Said tens models were also designed in CAD (FreeCAD; Jurgen Riegel, Germany) according to ISO 37:2017 [25] specification and sliced (Cura 4.2; Ultimaker, Netherlands) for 3D print. All materials were printed using fused deposition modelling technology (Ender-3; Creality, China) with 0.1 mm layer thickness, 100% infill density, 0.8 mm infill distance, and +45/−45 infill orientation to obtain unbiased results [26]. The designs were printed on a glass bed using PLA (PLAmax Fabbxible technology, Malaysia; *n* = 30) and PETG (Fabbxible technology, Malaysia;*n* = 30) according to manufacturers' recommended temperature and settings. The materials were printed in sets of five with each print session requiring 20 g (6.55 m) of print material. The process and resultant outcomes are illustrated in Figures 2(a), 2(b), 2(c), and 2(d).

#### 2.2.2. Test Models for Flexural Deformity and Break Force (Flex Models)

Silicone moulds were again fabricated to create PMMA samples (*n* = 30) in accordance with ISO 604:2002 [27, 28]. PLA (*n* = 30) and PETG (*n* = 30) samples were also designed in CAD following ISO 604:2002 and 3D printed (in sets of five) in the same manner as before.

### 2.3. Accuracy of 3D Print

Ten printed flex models were chosen from each material (PMMA, PLA, and PETG) using software-based randomisation (Random number generator; UX Apps, Russia). The length, width, and height of each sample were measured using a vernier caliper (Mitutoyo Absolute 500, Mitutoyo, Japan). Interrater reliability was first ensured between two researchers. Both researchers carried out linear measurements on 10 random specimens, and the resultant volumes were calculated. The geometric parameters were used to calculate the volume of each specimen and was subsequently used to calculate the root-mean-square (RMS) error in volumetric accuracy of each printed material. The formula was as follows:

(1) $$\begin{array}{c} {\text{RMS}}_{\text{mat}}=\frac{1}{\sqrt{n}}\cdot \sqrt{\sum_{i=0}^{n}{\left(r_{i}-o_{i}\right)}^{2}}, \end{array}$$

where RMS_mat_ = PMMA/PLA/PETG, *r*_*i*_ = ideal volume, and *o*_*i*_ = specimen volume.

### 2.4. Evaluation of Mass

All specimens were weighed using an electronic balance (BSA423S, Sartorius AG, Germany). Each sample was measured three times, and the average of the three measurements was recorded. The procedure was repeated again following immersion, and the mass changes were compared.

### 2.5. Evaluation of Density

The randomly selected flex models used for RMS were selected, and their mass and volume were used to calculate the density using the following formula:

(2) $$\begin{array}{c} \text{Density}=\frac{\text{Mass}}{\text{Volume}}. \end{array}$$

### 2.6. The Immersion Processes

The models (*n* = 180) were divided equally and randomly into five airtight containers. The models in container 1 were not immersed into any solutions and served as control for the postimmersion tests. Specimens within the remaining four containers were immersed into 250 ml [29] of the following fluid solutions: soybean oil (Buruh, Malaysia), carbonated soda (Coca-Cola Company, Malaysia Division), pasteurised milk (Dutch lady, Malaysia), and coffee (Nescafe classic, Malaysia). 2.5 g of coffee (measured using electronic balance) was mixed with 250 ml of purified hot water and allowed to cool down to room temperature prior to immersion [30, 31]. All the solutions were kept in room temperature, and the average daily temperatures were recorded (*Supplementary 2*). The soda and coffee were selected based on current reports of highest regional consumption [32, 33]. The immersion was carried out for 180 hr [34], and the solutions were changed and containers cleaned at every 24 -hr interval [35]. The specimens were rinsed under running tap water for 15 s while the solutions were being changed [35]. A new 250 ml soda can was used to refill the containers upon every 24-hr interval. After 180 hr, the materials were cleaned in running water, dried, and carried over for infrared spectroscopy and mechanical property testing.

### 2.7. Infrared Spectroscopy

Fourier-transform infrared spectroscopy (FTIR) was performed (Tensor 27, Netherlands) on one randomly selected (Random number generator; UX Apps, Russia) specimen from each material immersed into each solution. The device was set to record 32 sample scans at single channel transmittance of 4 cm^⁻1^ resolution. Data were recorded from 4,000  to 600 cm^⁻1^ wavelength on an interferogram of 14,220 points.

### 2.8. Plastic Deformity and Ultimate Tensile Stress Testing

The tens models were measured at three random points along its bridge for both breadth and height. The average value of each parameter was documented. Universal testing machine (Shimadzu, Universal testing machine, Japan) was used to measure the properties of the materials at 55 mm gauge length.

### 2.9. Flexural Deformity and Break Force Testing

The flex models were measured at three random points along its total length for both breadth and height. The average value of each parameter was documented. Universal testing machine (Shimadzu, Japan) was used to measure the properties of the materials, where the lower support was placed at 30 mm and force head set at 1 mm/min speed.

### 2.10. Statistical Analyses

All statistical analyses were carried out using SPSS v 26.0 (IBM Corp, America). Changes in mass and density across all five immersion media were evaluated through Shapiro–Wilk's test (*P* > 0.05) and by assessing the trends in the distribution histogram to confirm that data were mostly normally distributed. The normality of data generated from mechanical tests was assessed by examining histograms and bell-shaped curves. The robustness of the two-way ANOVA to mild deviations from normality was considered, given that other robustness factors, such as equal variance, limited outliers, and a balanced design for sample size, were adhered to. The differences in mass for each material in immersion solutions was independently evaluated using repeated measures analysis of variance. Density was analysed using one-way analysis of variance. Tensile and flexural test evaluations were carried out using multifactorial analysis of variance (*α* = 0.05).

## 3. Results

### 3.1. Reliability of Measure and Accuracy of 3D Print

Interrater reliability was measured for average absolute agreement at 0.844 (Cronbach's *α* = 0.887). The rootmean square of accuracy was found to be 297.684 for PMMA, 499.023 for PLA, and 539.326 for PETG. The test model volumes used to calculate the RMS are documented in *Supplementary 2*.

### 3.2. Evaluation of Mass

Prior to immersion, PLA (tens: 2.257 ± 0.088 and flex: 1.526 ± 0.055) and PETG (tens: 2.147 ± 0.327 and flex: 1.721 ± 0.071) had less mass than PMMA (tens: 3.116 ± 0.252 and flex: 1.996 ± 0.135). There were significant changes in mass following immersion for both tens models (within: *F* (df) = 1.367 (4), *P* = 0.253 and between: *F* (df) = 1.260 (4), *P* = 0.293) and flex models (within: *F* (df) = 0.258 (4), *P* = 0.904 and between: *F* (df) = 5.297 (4), *P* = 0.001). There were significant interactions between the materials and immersion media (tens: *F* (df) = 1.464 (8), *P* = 0.185 and flex: *F* (df) = , 4.832 (8), *P*  < 0.001) when mass was measured pre- and post-immersion. The results have been tabulated in Table 1.

### 3.3. Evaluation of Density

The mean density of PMMA, PLA, and PETG were 1.150 ± 0.154, 1.007 ± 0.052, and 1.163 ± 0.055, respectively. There were significant differences (*F* (df) = 7.613 (2), *P* = 0.002) present between the groups. Post hoc analysis (Tukey's) suggested significance differences among PMMA vs. PLA (*P* = 0.009) and PLA vs. PETG (*P* = 0.004).

### 3.4. Spectroscopic Evaluations

PMMA specimens immersed in oil showed a heightened peak at 2,800–3,000 cm^−1^ suggesting greater absorbance of CH_3_. All peaks for milk and caffeine media were substantially decreased. PLA specimens immersed in oil demonstrated substantially increased peaks at all intervals suggesting an increase in concentration of all functional groups. A new peak was observed at 3,300–3,350 cm^−1^ for PLA immersed in caffeine, suggesting absorbance of aliphatic primary amine. Soda solution and milk findings were unremarkable. PETG immersed in oil demonstrated a heightened peak at 2,900–2,922 cm^−1^. No other noteworthy changes were observed for PETG immersed in soda solution, milk solution, and caffeine solution. The graphs have been presented within the *Supplementary 2*.

### 3.5. Evaluation of Plastic Deformity and Ultimate Tensile Stress

There were no significant differences among the immersion media for both plastic deformity (*F* (df) = 1.700 (4), *P* = 0.159) and tensile load (*F* (df) = ⁻0.617 (4), *P* = 0.652). There was significant difference present in pairwise comparison of PMMA, PLA, and PETG for both plastic deformity (*F* (df) = 156.632 (2), *P*  < 0.001) and ultimate tensile stress (*F* (df) = 109.521 (2), *P*  < 0.001). Immersion media showed no significant interaction with materials for both plastic deformity (*F* (df) = 2.002 (8), *P* = 0.058) and ultimate tensile stress (*F* (df) = 0.706 (8), *P* = 0.685; Tables 2 and 3).

### 3.6. Evaluation of Flexural Deformity and Break Force

A significant difference (*F* (df) = 10.866 (4), *P*  < 0.001) was observed in flexural deformity among the immersion media. Pairwise comparison suggested no significant difference (*F* (df) = 2.693 (2), *P* = 0.074) among the materials. Immersion media showed significant interaction (*F* (df) = 4.984 (8), *P*  < 0.001) with materials (Table 4).

There were no significant differences in break force (*F* (df) = 0.441 (4), *P* = 0.779) among all media. There was, however, significant difference (*F* (df) = 319.785 (2), *P*  < 0.001) between PMMA, PLA, and PETG. Immersion media showed no significant interaction (*F* (df) = 1.370 (8), *P* = 0.223) with materials (Table 5).

## 4. Discussion

The current study aimed to explore the influence of the consumable liquids on the properties of 3D printable filaments after 180 hr of immersion. There were significant differences present in the parameters tested, and therefore, the null hypothesis was rejected.

PMMA demonstrated superior mechanical properties. This was due to the method of sample preparation. PLA and PETG were 3D printed at 0.1 mm layer thickness with +45/−45 infill orientation at a relatively lower print accuracy. PMMA was, however, packed into a custom mould. This resulted in higher volumes for PMMA samples with significant differences in density among the materials. It is important to note that PLA and PETG had 28%–32% less mass than PMMA which would theoretically translate to lighter (and more comfortable) prostheses.

Upon immersion, all three materials had a significant increase in mass following immersion with greater changes observed when specimens were immersed in milk and caffeine. A possible explanation for the changes in mass could be due to bacterial fermentation of the milk which took place during immersion. The staining properties of caffeine were observed in all samples with PMMA staining the most. PETG underwent the least amount of visual discoloration and was almost spectroscopically unchanged, which would suggest greater aesthetic resilience to food consumption.

It was found that the immersion media had no significant effect on plastic deformity, ultimate tensile stress, and break force with findings suggesting that PMMA was almost twice as strong as the 3D printable filaments. PETG performed marginally better than its PLA counterpart. The 3D models in the current study were printed at 0°–30° (horizontal) orientation which was found to produce the best tensile and flexural properties as opposed to 90° (vertical) build direction, which produced the lowest scores [36, 37, 38]. Further lowering layer thickness (below 0.1 mm) during 3D printing could have improved flexural properties in the current study [39]. Practically, however, using such layer thickness would also translate into longer printing times, which may not be suitable for immediate chairside prostheses fabrication.

The break force of PLA was most affected by all immersion media which may suggest that prostheses made of PLA will be more prone to fractures under regular use. The 3D printable materials when compared to PMMA were similarly resistant to flexural deformity and outperformed PMMA samples that were not submerged in any solutions. This was probably due to the increased hardness experienced by PMMA in dry conditions. This may indicate that while traditional PMMA-based prostheses need to be soaked overnight in water, PLA, or PETG filaments do not require such immersion, the material should be used to fabricate prostheses. Temperature was found to affect the tensile properties of both PLA and PETG with significant increases being found at above 30 °C [40, 41]. The current study was carried out at room temperature in a tropical climate. The temperature during the test was recorded between 25.5 and 27.5°C. This would agree with Ryokawa et al. [41] that perhaps PETG would provide better tensile properties if subjected intraorally at 37°.

In this study, it was observed that after exposure to various edible media, PMMA showed a reduction in plastic deformity and ultimate tensile stress. However, the tolerance to flexural deformity increased in both PMMA and PETG following exposure to edible liquids. This means that these materials become more resilient to angled occlusal forces during mastication, thereby tolerating the forces of chewing better than prostheses made from PLA. PLA prostheses demonstrated a lower break force, indicating a higher likelihood of fracturing. While PMMA showed increased resistance to breaking forces after extended exposure to edible liquids, this can be offset by the general process of aging, which reduces overall toughness [42]. Recent developments in fibre-reinforced PMMA can compensate for its lowered tensile properties [43]. Studies have also shown that polyamide-infused PMMAs produce transparent composites capable of overcoming their currently documented limitations. However, since their translation to dentistry is lacking, an in-depth evaluation of the aging process inside the mouth amidst exposure to edible liquids is needed [44].

The current study was limited to *in vitro* immersion testing at room temperature. To ensure practical applicability of the results, the authors digressed from the use of industrial 3D printers in favour of a low-cost commercial desktop 3D printer. The filaments were provided by a local manufacturer and was used to print the models at 0.1 mm layer thickness. As the method of PLA and PETG filament production vary globally among manufacturers, there may be minor differences in physical properties if the same study design was to be repeated with materials provided by a separate manufacturer. Of note, unlike PMMA, which is unaffected by thermocycling and staining, materials such as PLA and PETG may be prone to artificial ageing and is a topic that was not explored in the current investigation [45]. The longevity of such materials when subjected to live intraoral environments and salivary enzymes need to be evaluated to make further recommendations on their suitability as temporary dental prostheses.

## 5. Conclusion

Based on the findings of this study, the PLA and PETG 3D printable filaments tested that were printed at 0.1 mm thickness demonstrated inferior mechanical properties as opposed to PMMA when exposed to edible liquids. The study found PMMA and PETG became more resilient to forces simulating mastication, while PLA showed a higher fracture risk following exposure to edible liquids.

## Figures and Tables

**Table 1:** Repeated measures ANOVA for mass analysis.

Source	Sum square	Mean square	*F* stat (df)	*P* value
Within (tens models)^a^				
Time	0.317	0.317	7.476 (1)	0.008⁣^*∗*^
Time × immersion	0.232	0.058	1.367 (4)	0.253
Time × material	0.041	0.021	0.490 (2)	0.615
Time × immersion × material	0.166	0.021	0.489 (8)	0.860
Error (time)	3.178	0.042	—	—
Between (tens models)^a^				
Immersion	0.366	0.092	1.260 (4)	0.293
Material	31.563	15.782	217.060 (2)	<0.001⁣^*∗*^
Immersion × material	0.852	0.106	1.464 (8)	0.185
Error	5.453	0.073	—	—
Within (flex models)^b^				
Time	0.034	0.034	3.854 (1)	0.053
Time × immersion	0.009	0.002	0.258 (4)	0.904
Time × material	0.014	0.007	0.782 (2)	0.461
Time × immersion × material	0.005	0.001	0.074 (8)	> 0.999
Error (time)	0.657	0.009	—	—
Between (flex models)^b^				
Immersion	0.141	0.035	5.297 (4)	0.001⁣^*∗*^
Material	6.128	3.064	459.930 (2)	<0.001⁣^*∗*^
Immersion × material	0.258	0.032	4.832 (8)	<0.001⁣^*∗*^
Error	0.500	0.007	—	—

⁣^*∗*^Significant at 0.05; df, degree of freedom. Time = 2 levels (preimmersion and postimmersion). ^a^Tens models: pairwise comparison (materials): PMMA vs. PLA (*P*  < 0.001), PMMA vs. PETG (*P*  < 0.001), and PLA vs. PETG (*P* = 0.133). Pairwise comparison (immersion methods): no immersion vs. oil (*P* = 0.859), no immersion vs. soda (*P* = 0.603), no immersion vs. milk (*P* = 0.203), no immersion vs. caffeine (*P* = 0.895), oil vs. soda (P = 0.991), oil vs. milk (*P* = 0.763), oil vs. caffeine (*P* > 0.999), soda vs. milk (*P* = 0.950), soda vs. caffeine (*P* = 0.983), and milk vs. caffeine (*P* = 0.713). ^b^Flex models: pairwise comparison (materials): PMMA vs. PLA (*P*  < 0.001), PMMA vs. PETG (*P*  < 0.001), and PLA vs. PETG (*P*  < 0.001). Pairwise comparison (immersion methods): no immersion vs. oil (*P* = 0.712), no immersion vs. soda (*P* = 0.219), no immersion vs. milk (*P* = 0.026), no immersion vs. caffeine (*P* = 0.001), oil vs. soda (*P* = 0.910), oil vs. milk (*P* = 0.392), oil vs. caffeine (*P* = 0.031), soda vs. milk (*P* = 0.888), soda vs. caffeine (*P* = 0.224), and milk vs. caffeine (*P* = 0.754).

**Table 2:** Multifactorial ANOVA for plastic deformity (N/mm^2^) analysis.

Dependent variable: plastic deformation Factor: (1) Immersion media: *F* stat (df) = 1.700 (4), *P* value = 0.159 No immersion: mean (SD) = 1,697.642 (795.083) Oil: mean (SD) = 1,701.750 (654.633) Soda: mean (SD) = 1,538.508 (489.198) Milk: mean (SD) = 1,646.288 (701.976) Caffeine: mean (SD) = 1,498.852 (624.197) (2) Materials: *F* stat (df) = 156.632 (2), *P* value <0.001⁣^*∗*^			
	PMMA	PLA	PETG

	Mean (SD)	Mean (SD)	Mean (SD)
No immersion	2,666.058 (492.572)	1,223.822 (116.422)	1,203.047 (452.547)
Oil	2,501.647 (430.745)	1,294.625 (94.736)	1,308.978 (332.732)
Soda	2,036.885 (478.581)	1,378.548 (54.442)	1,200.091 (339.818)
Milk	2,555.482 (296.930)	1,319.295 (122.907)	1,064.087 (212.434)
Caffeine	2,293.068 (244.417)	1,278.328 (176.299)	925.161 (153.799)

Pairwise comparison			

(1) Immersion media	*P* value	(2) Materials	*P* value

No immersion—oil	> 0.999	PMMA–PLA	<0.001⁣^*∗*^
No immersion—soda	> 0.999		
No immersion—milk	> 0.999		

No immersion—caffeine	0.527	PMMA–PETG	<0.001⁣^*∗*^
Oil—soda	> 0.999		
Oil—milk	> 0.999		
Oil—caffeine	0.481		

Soda—milk	> 0.999	PLA–PETG	0.138
Soda—caffeine	> 0.999		
Milk—caffeine	> 0.999		

Interaction effect: immersion media vs. materials. *F* stat (df) = 2.002 (8), *P* value = 0.058. ⁣^*∗*^Significant at <0.05. SD, standard deviation.

**Table 3:** Multifactorial ANOVA for ultimate tensile stress (*N*) analysis.

Dependent variable: ultimate tensile stress Factor: (1) Immersion media: *F* stat (df) = 0.617 (4), *P* value = 0.652 No immersion: mean (SD) = 453.613 (229.275) Oil: mean (SD) = 457.502 (170.120) Soda: mean (SD) = 449.900 (203.068) Milk: mean (SD) = 412.857 (184.846) Caffeine: mean (SD) = 426.906 (188.548) (2) Materials: *F*- stat (df) = 109.521 (2), *P* value <0.001⁣^*∗*^			
	PMMA	PLA	PETG

	Mean (SD)	Mean (SD)	Mean (SD)
No immersion	731.243 (127.877)	346.924 (23.402)	282.672 (143.558)
Oil	660.436 (118.209)	357.859 (29.131)	354.211 (97.167)
Soda	688.376 (147.262)	349.279 (21.211)	312.045 (121.937)
Milk	618.706 (173.286)	356.080 (10.100)	263.784 (68.238)
Caffeine	639.595 (111.323)	382.629 (43.720)	258.494 (126.074)

Pairwise comparison			

(1) Immersion media	*P* value	(2) Materials	*P* value

No immersion—oil	> 0.999	PMMA–PLA	<0.001⁣^*∗*^
No immersion—soda	> 0.999		
No immersion—milk	> 0.999		

No immersion—caffeine	> 0.999	PMMA–PETG	<0.001⁣^*∗*^
Oil—soda	> 0.999		
Oil—milk	> 0.999		
Oil—caffeine	> 0.999		

Soda—milk	> 0.999	PLA–PETG	0.059
Soda—caffeine	> 0.999		
Milk—caffeine	> 0.999		

Interaction effect: immersion media vs. materials. *F* stat (df) = 0.706 (8), *P* value = 0.685. ⁣^*∗*^Significant at <0.05. SD, standard deviation.

**Table 4:** Multifactorial ANOVA for flexural deformity analysis (N/mm^2^).

Dependent variable: flexural deformity Factor: (1) Immersion media: *F* stat (df) = 10.866 (4), *P* value <0.001⁣^*∗*^ No immersion: mean (SD) = 1113.729 (456.729) Oil: mean (SD) = 1530.370 (290.148) Soda: mean (SD) = 1636.775 (235.995) Milk: mean (SD) = 1571.278 (246.454) Caffeine: mean (SD) = 1486.294 (292.442) (1) Materials: *F* stat (df) = 2.693 (2), *P* value = 0.074			
	PMMA	PLA	PETG

	Mean (SD)	Mean (SD)	Mean (SD)
No immersion	601.438 (376.113)	1410.512 (61.418)	1329.239 (296.063)
Oil	1600.388 (367.097)	1400.690 (283.405)	1590.032 (202.037)
Soda	1796.777 (332.022)	1549.545 (148.941)	1564.003 (103.590)
Milk	1600.702 (398.722)	1457.302 (66.099)	1655.830 (134.016)
Caffeine	1452.949 (470.203)	1354.460 (60.656)	1651.473 (105.234)

Pairwise comparison			

(1) Immersion media	*P* value	(2) Materials	*P* value

No immersion—oil	<0.001⁣^*∗*^	PMMA–PLA	> 0.999
No immersion—soda	<0.001⁣^*∗*^		
No immersion—milk	<0.001⁣^*∗*^		

No immersion—caffeine	0.001⁣^*∗*^	PMMA–PETG	0.101
Oil—soda	> 0.999		
Oil—milk	> 0.999		
Oil—caffeine	> 0.999		

Soda—milk	> 0.999	PLA–PETG	0.223
Soda—caffeine	0.920		
Milk—caffeine	> 0.999		

Interaction effect: immersion media vs. materials. *F* stat (df) = 4.984 (8), *P* value <0.001⁣^*∗*^. ⁣^*∗*^Significant at <0.05. SD, standard deviation.

**Table 5:** Multifactorial ANOVA for break force (*N*) analysis.

Dependent variable: break force Factor: (1) Immersion media: *F* stat (df) = .441 (4), *P* value = 0.779 No immersion: mean (SD) = 123.318 (51.687) Oil: mean (SD) = 121.929 (58.774) Soda: mean (SD) = 122.769 (67.664) Milk: mean (SD) = 124.202 (64.974) Caffeine: mean (SD) = 130.429 (72.201) (2) Materials: *F* stat (df) = 319.785 (2), *P* value <0.001⁣^*∗*^			
	PMMA	PLA	PETG

	Mean (SD)	Mean (SD)	Mean (SD)
No immersion	188.220 (28.343)	76.041 (8.207)	105.694 (10.146)
Oil	197.266 (29.724)	73.292 (9.579)	95.228 (16.266)
Soda	206.115 (46.756)	64.797 (6.491)	97.395 (14.039)
Milk	205.503 (21.148)	57.866 (10.374)	109.235 (17.823)
Caffeine	222.961 (37.119)	66.540 (8.352)	101.785 (10.921)

Pairwise comparison			

(1) Immersion media	*P* value	(2) Materials	*P* value

No immersion—oil	> 0.999	PMMA–PLA	<0.001⁣^*∗*^
No immersion—soda	> 0.999		
No immersion—milk	> 0.999		

No immersion—caffeine	> 0.999	PMMA–PETG	<0.001⁣^*∗*^
Oil—soda	> 0.999		
Oil—milk	> 0.999		
Oil—caffeine	> 0.999		

Soda—milk	> 0.999	PLA–PETG	<0.001⁣^*∗*^
Soda—caffeine	> 0.999		
Milk—caffeine	> 0.999		

Interaction effect: immersion media vs. materials. *F* stat (df) = 1.370 (8), *P* value = 0.223. ⁣^*∗*^Significant at <0.05. SD, standard deviation.

## Data Availability

The data that support the findings of this study are available in the supplementary material of this article.

## Supplementary Materials

Supplementary 1 The schematics and 3D printable information of the specimens used in the current study. All metadata generated from mass, volume, density, temperate, spectroscopy, tensile and flexural stress tests have been combined and provided as a single file.

Supplementary 2 Consolidated appendix.
