# Supplementary material for: In Vitro Characterisation of 3D Printable Filaments Subjected to Edible Liquids: An Analysis of Fused Deposition Modelling for Intraoral Applicability
Source: Int J Dent. 2024 Aug 30;2024:2118412. doi: 10.1155/2024/2118412 (PMC11479776; doi:10.1155/2024/2118412)
Supplement: Supplementary 2 — Consolidated appendix. [file 2118412.f2.pdf]

SUPPLEMENTARY MATERIAL 2: CONSOLIDATED APPENDIX

Contents

1. Inter-rater reliability results measurements........ 2
2. Measurements for root mean square accuracy of volume 3
3. Mass change data 4
4. Density data 6
5. Temperature data 7
6. FTIR Spectroscopy data 8
7. Tensile strength data 20
8. Flexural strength data 23

1. Inter-rater reliability results measurements

Table 1.1: Inter-rater reliability results (in mm)

No.	Rater 1				Rater 2			
	Length	*Width*	*Height*	***Volume (mm³)***	*Length*	*Width*	*Height*	***Volume (mm³)***
1	49.57	9.94	3.44	1694.977	49.65	9.94	3.84	1895.120
2	49.21	9.89	3.95	1922.413	49.83	10.03	3.71	1854.539
3	49.61	10.05	3.48	1735.060	49.81	10.30	3.58	1788.547
4	49.85	10.11	3.70	1864.739	49.99	10.35	4.10	2121.326
5	48.95	9.89	3.71	1796.068	49.11	9.92	3.78	1841.507
6	49.76	10.09	3.47	1742.212	50.03	10.12	3.50	1772.063
7	49.83	10.12	3.97	2001.990	49.95	10.34	3.99	2060.767
8	49.58	9.99	3.29	1629.551	49.43	10.01	3.41	1687.248
9	49.15	9.84	3.25	1571.817	49.46	9.90	3.28	1606.065
10	49.52	10.06	3.46	1723.672	49.78	10.42	3.36	1742.857

2. Measurements for root mean square accuracy of volume

Table 2.1: Volumes (in mm^3) obtained from specimen for RMS accuracy assessment

No.	***PMMA***	***PLA***	***PETG***
1	1511.863	1546.505	1466.156
2	1750.391	1560.001	1448.563
3	1957.818	1530.708	1485.949
4	1636.033	1540.150	1442.358
5	1785.529	1442.350	1501.973
6	1916.759	1475.986	1452.124
7	1781.585	1427.647	1425.842
8	1930.090	1449.086	1460.418
9	1628.616	1516.759	1497.157
10	1544.046	1542.714	1432.038

3. Mass change data

Table 3.1: Mass (in grams) changes in specimen undergoing no immersion (n=36)

No.	PMMA		PLA		PETG	
	Before	*After*	*Before*	*After*	*Before*	*After*
1	3.255	3.253	2.201	2.202	2.110	2.218
2	2.928	2.922	2.351	2.351	2.343	2.346
3	2.920	2.929	2.310	2.311	2.553	2.556
4	3.092	3.094	2.045	2.046	2.101	2.101
5	3.253	3.255	2.330	2.332	1.354	1.356
6	3.048	3.050	2.188	2.190	2.215	2.122
7	1.793	1.794	1.609	1.609	1.743	1.745
8	2.075	2.077	1.540	1.541	1.751	1.752
9	1.844	1.844	1.460	1.463	1.649	1.651
10	1.864	1.867	1.497	1.499	1.767	1.768
11	1.979	1.978	1.523	1.525	1.662	1.663
12	1.976	1.977	1.483	1.485	1.741	1.743

Table 3.2: Mass (in grams) changes in PMMA specimen following Immersion (n=48)

No.	Cooking oil		Soda		Milk		Caffeine	
	Before	*After*	*Before*	*After*	*Before*	*After*	*Before*	*After*
1	2.471	3.005	3.638	3.653	3.229	3.232	3.172	3.187
2	2.992	3.238	3.126	3.106	3.045	3.060	2.881	2.897
3	2.309	2.476	3.321	3.333	3.211	3.226	3.042	3.089
4	3.138	3.147	3.162	3.135	3.333	3.350	3.346	3.380
5	3.226	3.323	3.126	3.139	3.218	3.245	3.361	3.363
6	3.326	3.335	3.092	3.174	3.147	3.160	3.073	3.055
7	2.020	1.834	2.210	2.219	2.111	2.122	2.198	2.210
8	1.885	1.890	2.048	2.059	1.833	1.843	1.883	1.896
9	1.743	1.749	2.077	2.087	2.090	2.100	2.203	2.215
10	1.826	2.031	1.951	1.962	2.023	2.036	2.286	2.298
11	1.941	1.949	1.923	1.935	1.996	2.007	2.009	2.020
12	1.967	1.971	2.055	2.064	2.163	2.176	1.923	1.933

Table 3.3: Mass (in grams) changes in PLA specimen following Immersion (n=48)

No.	Cooking oil		Soda		Milk		Caffeine	
	Before	*After*	*Before*	*After*	*Before*	*After*	*Before*	*After*
1	2.173	2.334	2.341	2.449	2.278	2.418	2.272	2.350
2	2.197	2.266	2.203	2.221	2.357	2.631	2.301	2.349
3	2.203	2.350	2.215	2.252	2.343	2.509	2.363	2.332
4	2.346	2.454	2.157	2.204	2.369	2.396	2.403	2.543
5	2.116	2.283	2.148	2.254	2.280	2.472	2.317	2.491
6	2.255	2.330	2.206	2.234	2.264	2.566	2.179	2.268
7	1.513	1.600	1.505	1.594	1.467	1.657	1.543	1.659
8	1.490	1.540	1.506	1.523	1.552	1.601	1.578	1.636
9	1.597	1.580	1.439	1.468	1.549	1.548	1.595	1.639
10	1.620	1.660	1.513	1.540	1.491	1.523	1.465	1.476
11	1.473	1.664	1.625	1.683	1.426	1.549	1.540	1.654
12	1.593	1.639	1.483	1.551	1.536	1.609	1.576	1.586

Table 3.4: Mass (in grams) changes in PETG specimen following Immersion (n=48)

No.	Cooking oil		Soda		Milk		Caffeine	
	Before	*After*	*Before*	*After*	*Before*	*After*	*Before*	*After*
1	2.216	2.473	2.272	2.345	2.116	2.587	2.167	2.184
2	2.096	2.234	2.242	2.355	2.036	2.438	2.537	2.566
3	2.302	2.308	2.263	2.321	2.227	2.579	2.167	2.248
4	2.701	2.781	2.329	2.287	2.212	2.582	1.403	1.428
5	2.148	2.327	2.617	2.635	1.480	1.897	2.178	2.210
6	2.217	2.399	1.482	1.493	2.237	2.462	2.084	2.111
7	1.803	1.806	1.699	1.712	1.849	1.862	1.814	1.832
8	1.787	1.854	1.568	1.604	1.686	1.702	1.732	1.734
9	1.786	1.829	1.634	1.652	1.782	1.763	1.807	1.820
10	1.681	1.758	1.761	1.783	1.714	1.769	1.733	1.753
11	1.629	1.675	1.795	1.823	1.647	1.718	1.574	1.587
12	1.689	1.742	1.673	1.687	1.750	1.798	1.722	1.747

4. Density data

Table 4.1: Density analysis of materials

Material	Volume (V)	Mass (mg)	Density
PMMA	1511.863	1844	1.220
PMMA	1750.391	1864	1.065
PMMA	1957.818	1979	1.011
PMMA	1636.033	2020	1.235
PMMA	1785.529	1885	1.056
PMMA	1916.759	1743	0.909
PMMA	1781.585	2210	1.240
PMMA	1930.090	2048	1.061
PMMA	1628.616	2077	1.275
PMMA	1544.046	2203	1.427
PLA	1546.505	1497	0.968
PLA	1560.001	1523	0.976
PLA	1530.708	1483	0.969
PLA	1540.150	1620	1.052
PLA	1442.350	1473	1.021
PLA	1475.986	1593	1.079
PLA	1427.647	1426	0.909
PLA	1449.086	1536	1.060
PLA	1516.759	1540	1.015
PLA	1542.714	1576	1.021
PETG	1466.156	1767	1.205
PETG	1448.563	1662	1.147
PETG	1485.949	1741	1.172
PETG	1442.358	1786	1.238
PETG	1501.973	1681	1.120
PETG	1452.124	1629	1.122
PETG	1425.842	1782	1.250
PETG	1460.418	1714	1.174
PETG	1497.157	1647	1.100
PETG	1432.038	1574	1.099

5. Temperature data

Table 5.1: Daily average temperature analysis

**Hour**	**Average temperature (℃)**
0-24	27.5
25-48	26.5
49-72	25.5
73-96	27.5
97-120	27
121-144	26.5
145-168	26
169-180	27

6. FTIR Spectroscopy data

6.1: PMMA; No immersion Vs Oil media spectrum

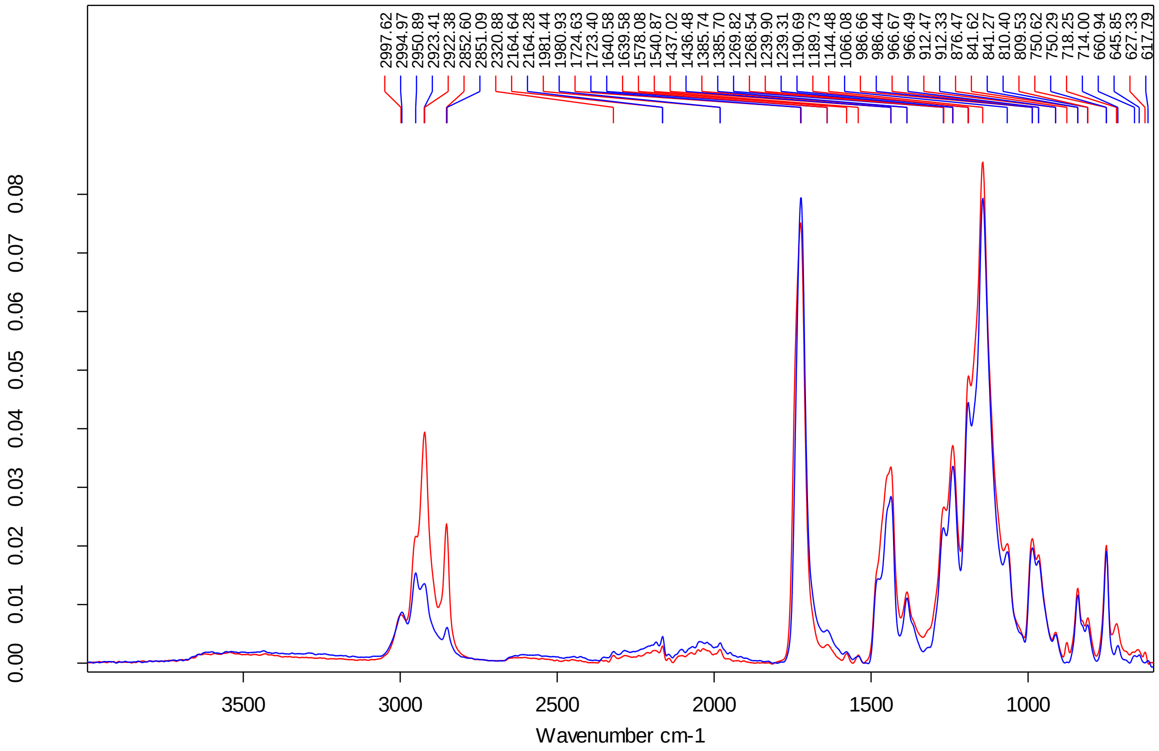

C:\OPUS_7.0.122\MEAS\aparna immersion FTIR\No immersion ACRYLIC.0 No immersion ACRYLIC	20/12/2020
C:\OPUS_7.0.122\MEAS\aparna immersion FTIR\Oil ACRYLIC.1 Oil ACRYLIC	20/12/2020

6.2: PMMA; No immersion Vs Soda media spectrum

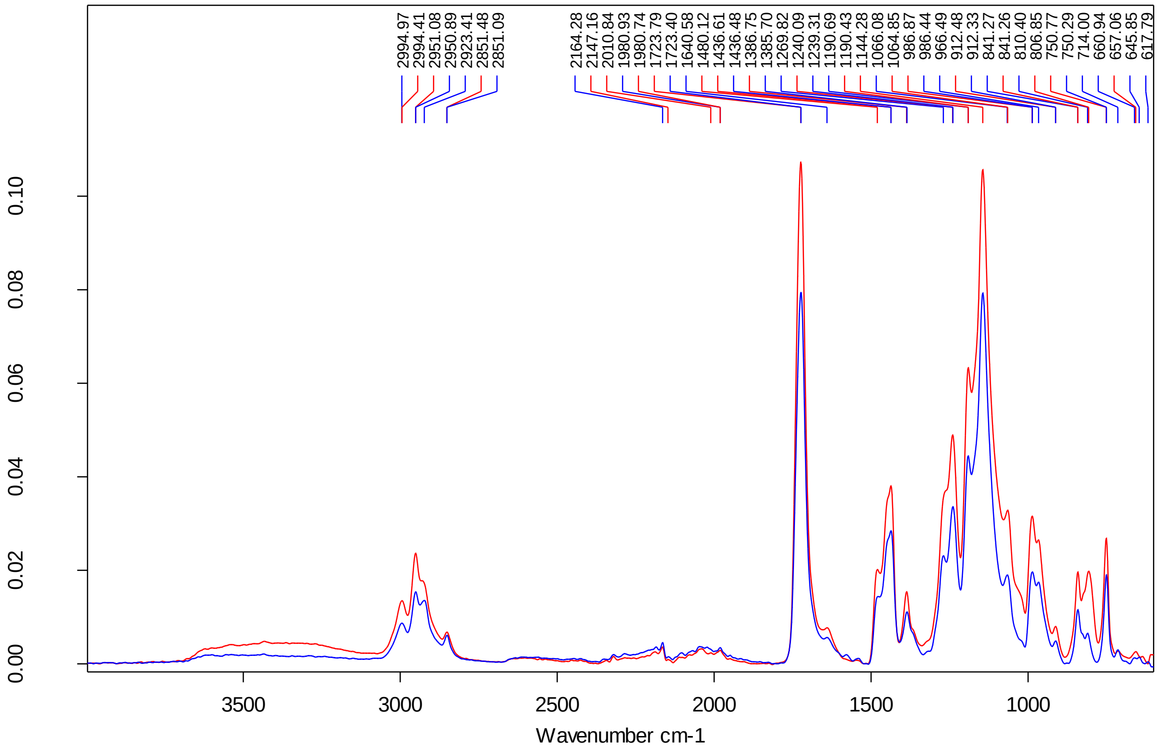

C:\OPUS_7.0.122\MEAS\aparna immersion FTIR\No immersion ACRYLIC.0 No immersion ACRYLIC	20/12/2020
C:\OPUS_7.0.122\MEAS\aparna immersion FTIR\Soda ACRYLIC.0 Soda ACRYLIC	20/12/2020

6.3: PMMA; No immersion Vs Milk media spectrum

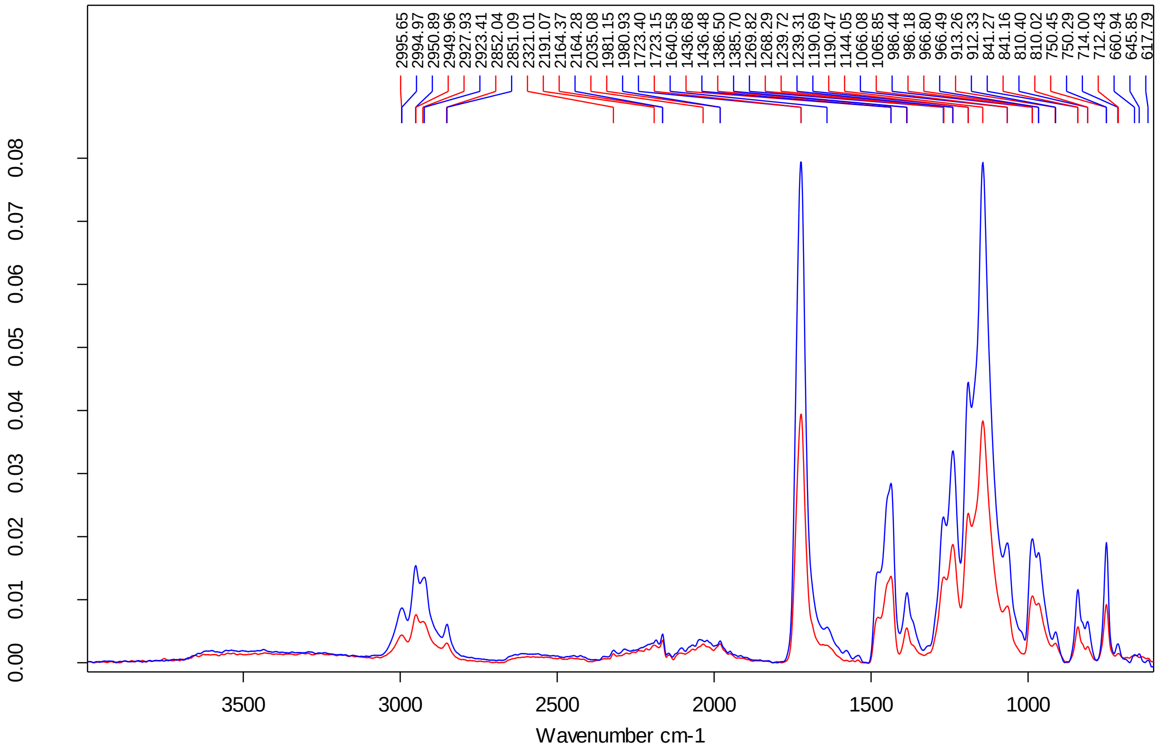

C:\OPUS_7.0.122\MEAS\aparna immersion FTIR\No immersion ACRYLIC.0 No immersion ACRYLIC		20/12/2020
C:\OPUS_7.0.122\MEAS\aparna immersion FTIR\Milk ACRYLIC.0 Milk ACRYLIC		20/12/2020

6.4: PMMA; No immersion Vs Caffeine media spectrum

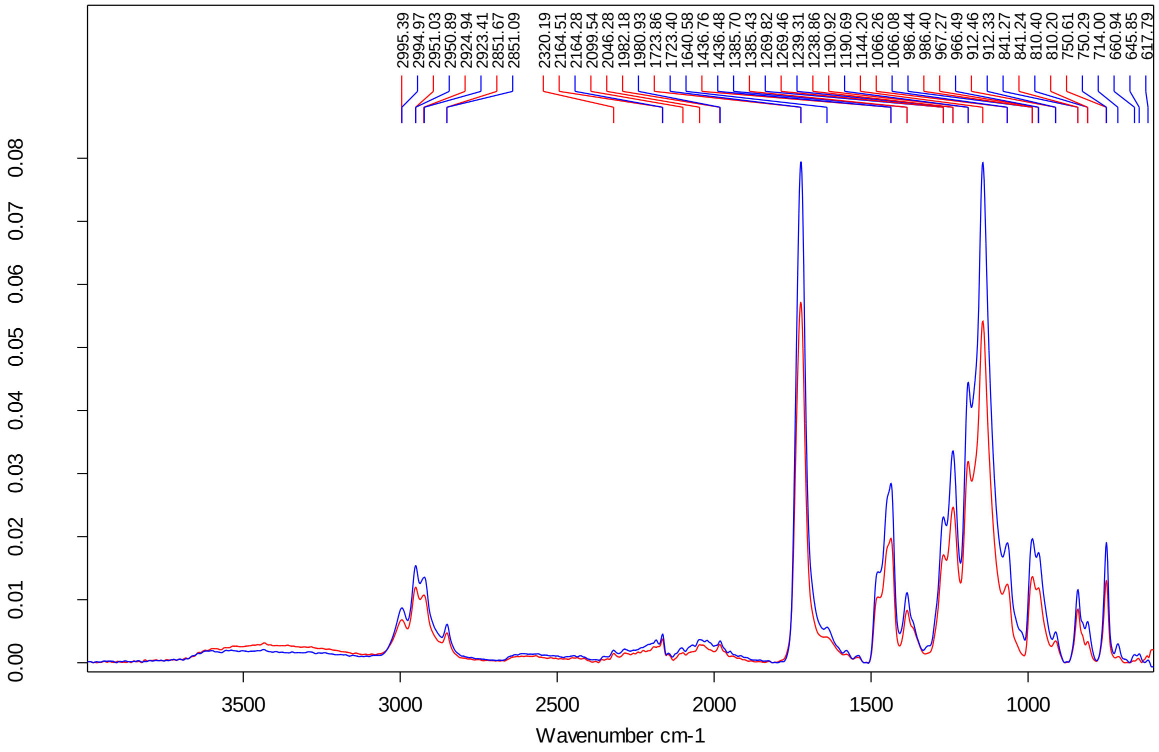

C:\OPUS_7.0.122\MEAS\aparna immersion FTIR\No immersion ACRYLIC.0 No immersion ACRYLIC		20/12/2020
C:\OPUS_7.0.122\MEAS\aparna immersion FTIR\Caffeine ACRYLIC.0 Caffeine ACRYLIC		20/12/2020

6.5: PLA; No immersion Vs Oil spectrum

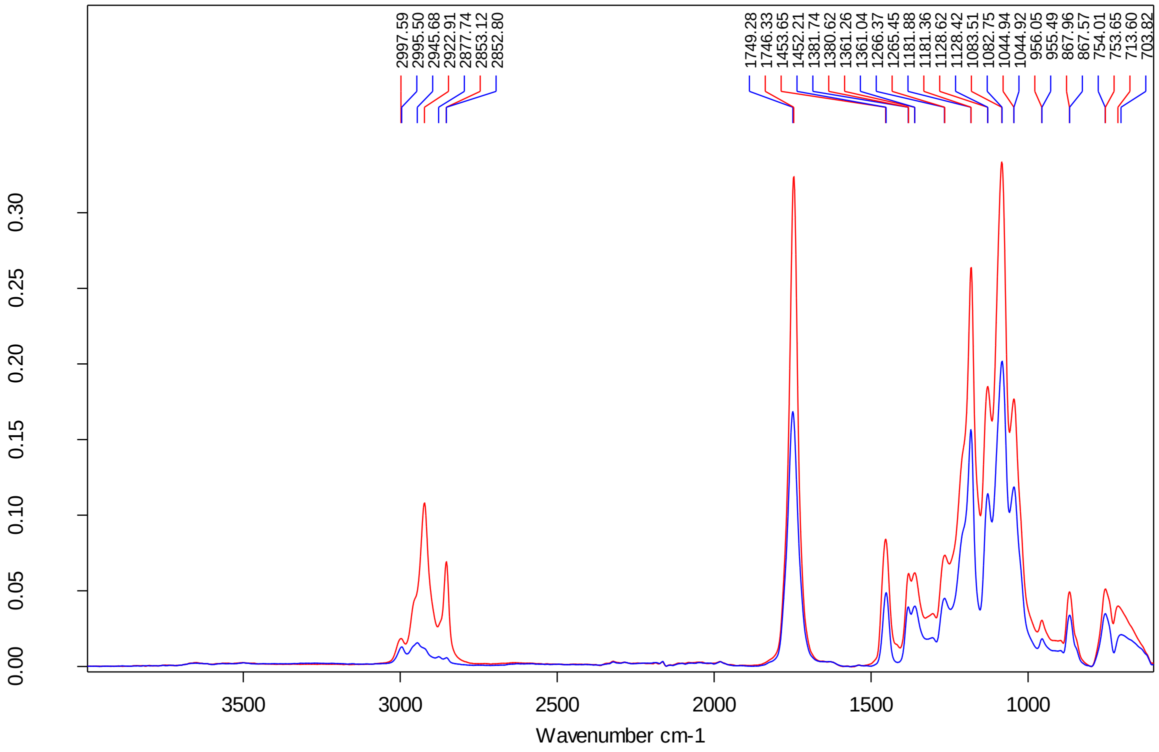

C:\OPUS_7.0.122\MEAS\aparna immersion FTIR\No immersion PLA.0 No immersion PLA		20/12/2020
C:\OPUS_7.0.122\MEAS\aparna immersion FTIR\oil PLA.1 oil PLA		20/12/2020

6.6: PLA; No immersion Vs Soda media spectrum

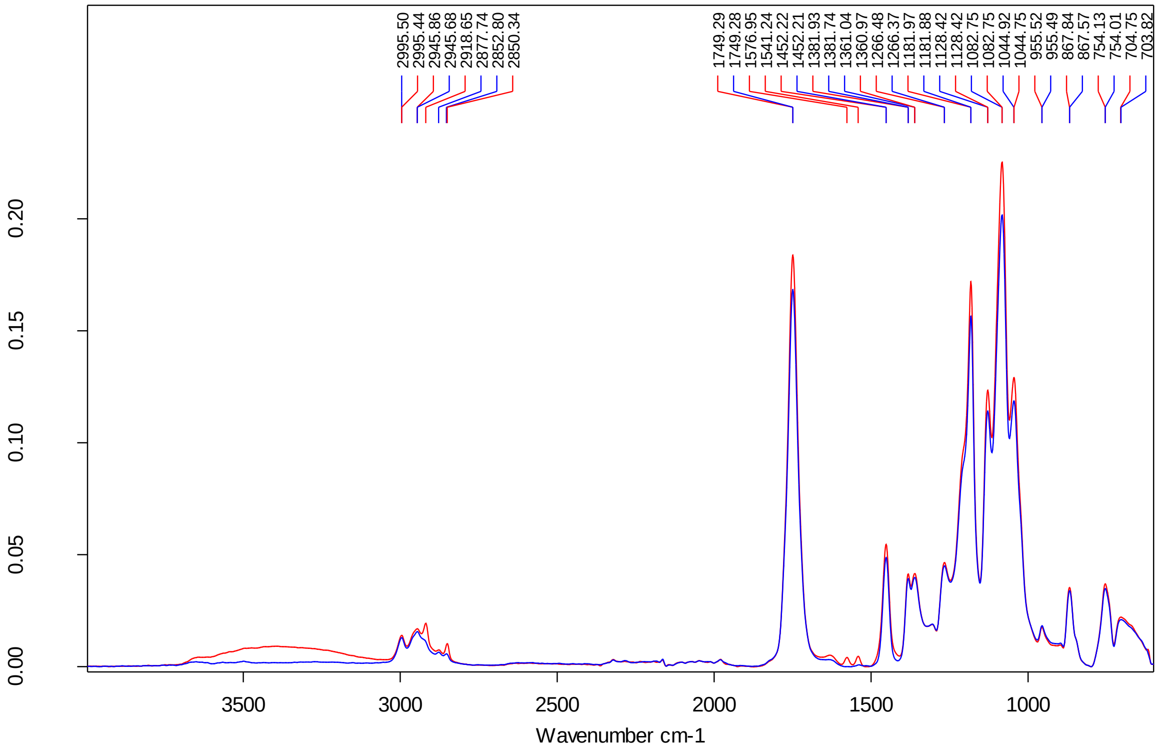

C:\OPUS_7.0.122\MEAS\aparna immersion FTIR\No immersion PLA.0 No immersion PLA	20/12/2020
C:\OPUS_7.0.122\MEAS\aparna immersion FTIR\soda PLA.0 soda PLA	20/12/2020

6.7: PLA; No immersion Vs Milk media spectrum

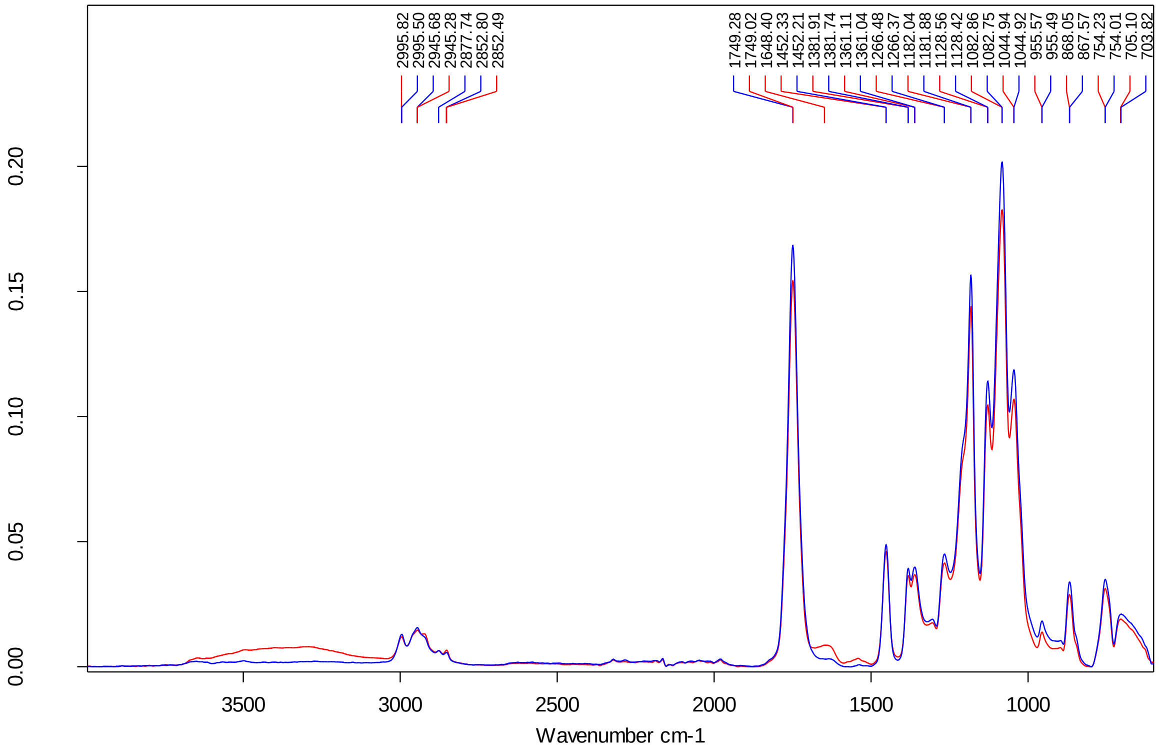

C:\OPUS_7.0.122\MEAS\aparna immersion FTIR\No immersion PLA.0	No immersion PLA	20/12/2020
C:\OPUS_7.0.122\MEAS\aparna immersion FTIR\milk PLA.0	milk PLA	20/12/2020

6.8: PLA; No immersion Vs Caffeine media spectrum

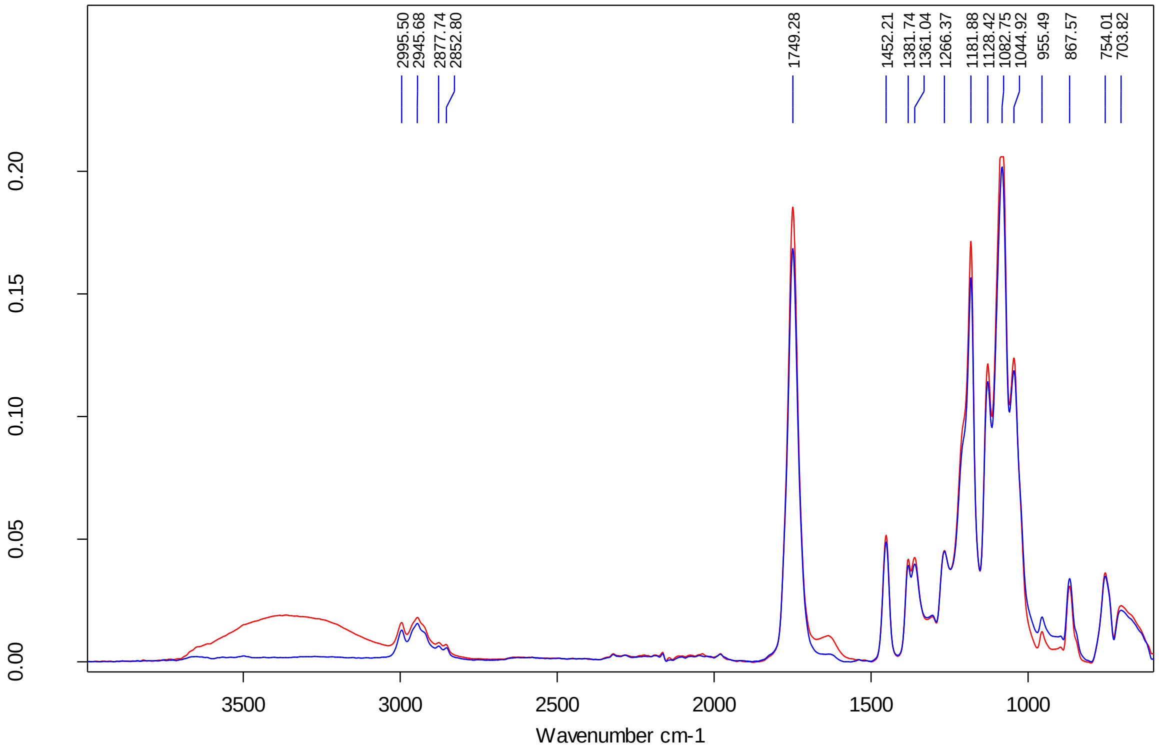

C:\OPUS_7.0.122\MEAS\aparna immersion FTIR\No immersion PLA.0	No immersion PLA	20/12/2020
C:\OPUS_7.0.122\MEAS\aparna immersion FTIR\caffeine PLA.0	caffeine PLA	20/12/2020

6.9: PETG; No immersion Vs Oil media spectrum

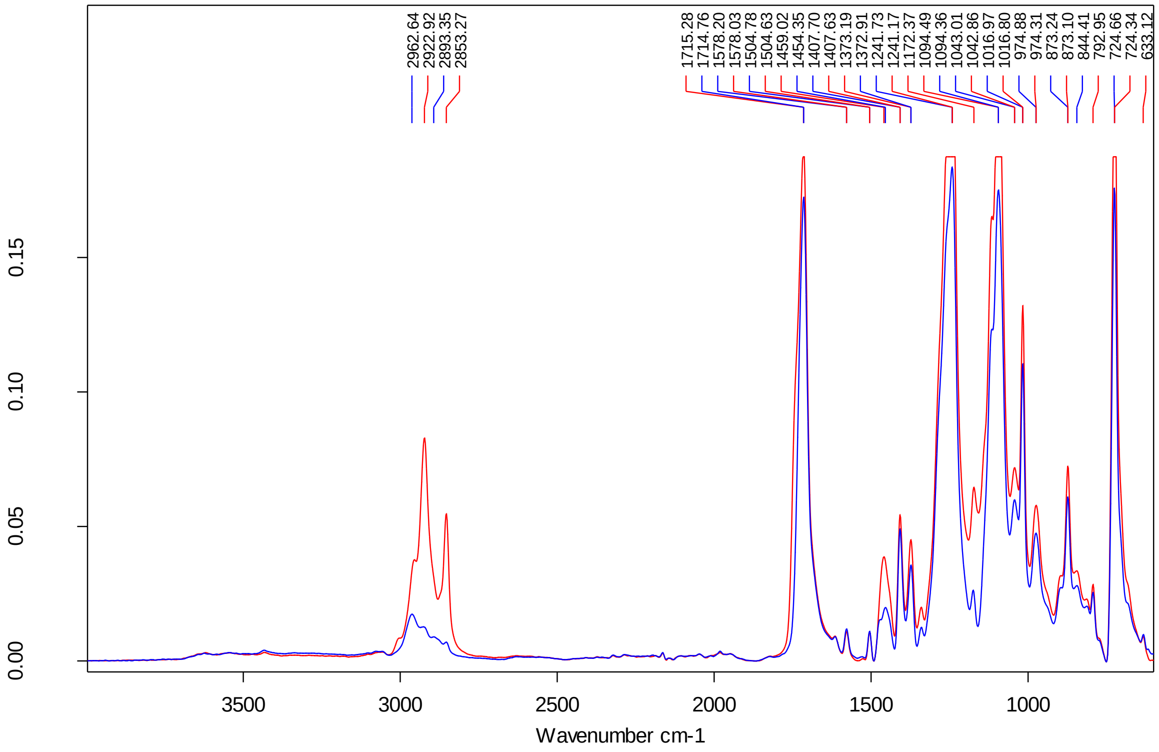

C:\OPUS_7.0.122\MEAS\aparna immersion FTIR\no immersion 1.0	no immersion 1	20/12/2020
C:\OPUS_7.0.122\MEAS\aparna immersion FTIR\oil 1 PETG.0	oil 1 PETG	20/12/2020

6.10: PETG; No immersion Vs Soda media spectrum

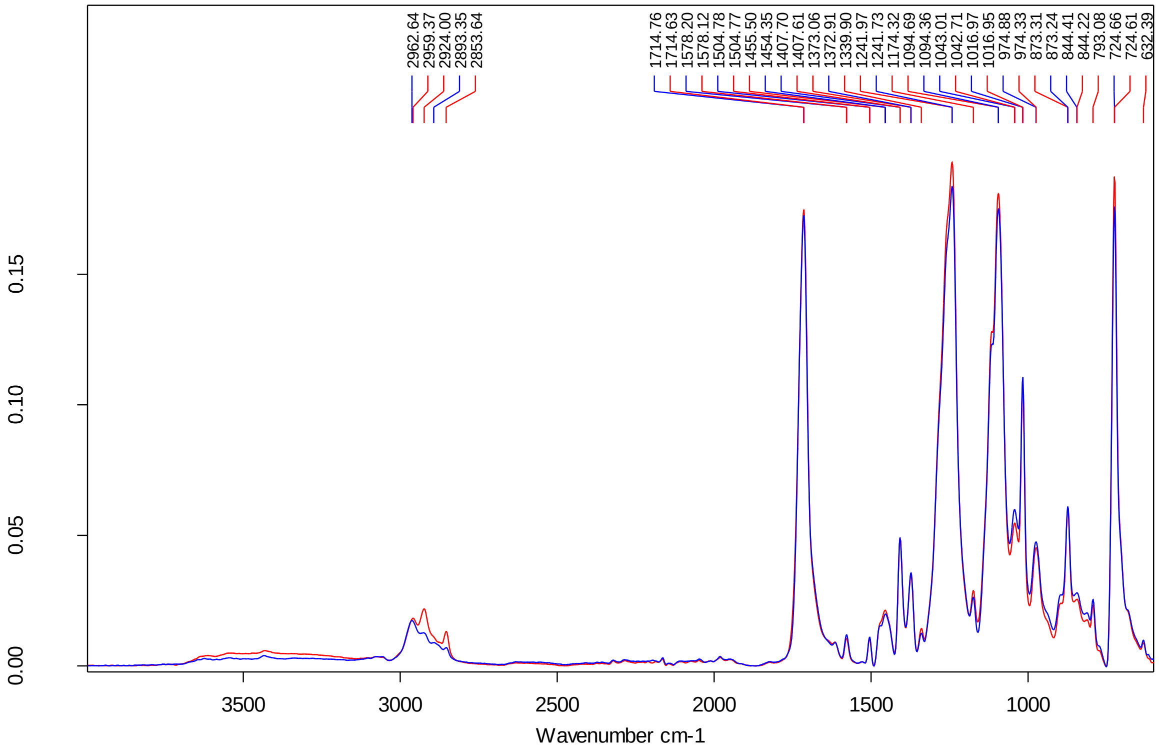

C:\OPUS_7.0.122\MEAS\aparna immersion FTIR\no immersion 1.0 no immersion 1		20/12/2020
C:\OPUS_7.0.122\MEAS\aparna immersion FTIR\soda 1 PETG.0 soda 1 PETG		20/12/2020

6.11: PETG; No immersion Vs Milk media spectrum

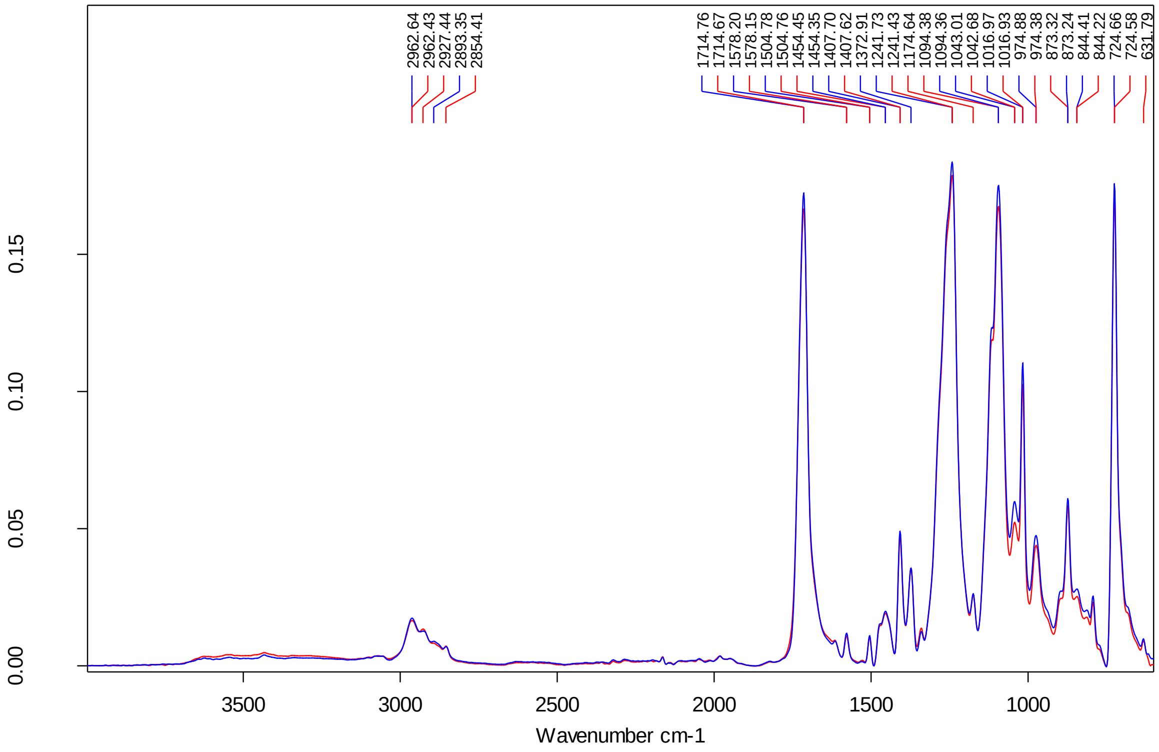

C:\OPUS_7.0.122\MEAS\aparna immersion FTIR\no immersion 1.0 no immersion 1	20/12/2020
C:\OPUS_7.0.122\MEAS\aparna immersion FTIR\milk 1 PETG.0 milk 1 PETG	20/12/2020

6.12: PETG; No immersion Vs Caffeine media spectrum

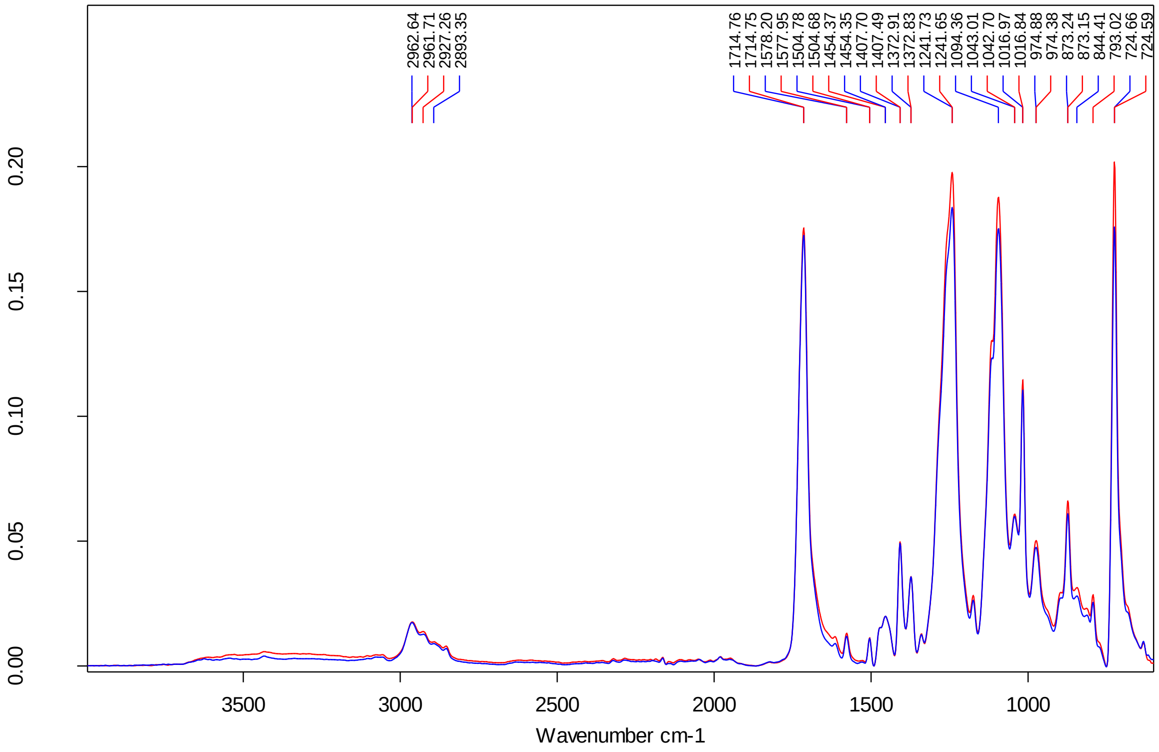

C:\OPUS_7.0.122\MEAS\aparna immersion FTIR\no immersion 1.0	no immersion 1	20/12/2020
C:\OPUS_7.0.122\MEAS\aparna immersion FTIR\caffeine 1 PETG.0	caffeine 1 PETG	20/12/2020

7. Tensile strength data

Table 7.1: Tensile strength linear measurements (in mm) for PMMA (n=30)

	No immersion		Cooking oil		Soda		Milk		Caffeine	
	breadth	*Height*	*breadth*	*Height*	*breadth*	*Height*	*breadth*	*Height*	*breadth*	*Height*
1	3.85	4.03	3.95	3.99	4.24	4.02	3.92	4.10	4.01	4.18
2	3.89	3.92	4.03	4.20	4.39	4.18	3.93	3.96	3.85	4.01
3	4.01	4.08	3.91	4.00	3.92	4.23	3.90	3.99	3.94	4.14
4	3.96	3.94	4.05	4.18	4.01	4.12	3.98	4.16	3.94	4.46
5	3.90	3.98	4.07	4.15	3.95	4.25	3.98	4.08	3.92	3.81
6	4.01	4.26	3.92	4.18	3.94	4.08	4.07	4.33	4.00	4.08

Table 7.2: Tensile strength linear measurements (in mm) for PLA (n=30)

	No immersion		Cooking oil		Soda		Milk		Caffeine	
	breadth	*Height*	*breadth*	*Height*	*breadth*	*Height*	*breadth*	*Height*	*breadth*	*Height*
1	5.01	3.19	4.89	3.05	4.95	2.98	4.96	3.07	4.90	3.31
2	4.95	3.14	5.00	2.92	4.90	3.10	4.89	2.98	4.90	3.12
3	4.96	2.95	4.98	2.99	4.94	2.87	4.89	3.03	4.93	3.34
4	4.93	2.99	4.92	2.99	4.94	2.90	5.08	3.10	4.87	3.23
5	5.06	2.82	4.98	2.86	4.97	2.89	4.93	2.98	4.85	3.16
6	4.90	3.12	4.87	3.11	4.93	2.88	4.89	3.15	4.90	3.21

Table 7.3: Tensile strength linear measurements (in mm) for PETG (n=30)

	No immersion		Cooking oil		Soda		Milk		Caffeine	
	breadth	*Height*	*breadth*	*Height*	*breadth*	*Height*	*breadth*	*Height*	*breadth*	*Height*
1	4.74	3.35	4.88	3.16	4.96	3.10	4.52	2.70	4.48	2.70
2	4.87	2.92	4.63	3.31	4.59	3.40	4.71	3.16	4.66	3.33
3	4.78	3.46	4.58	3.12	4.59	2.84	4.70	3.27	4.70	3.33

4	4.47	2.60	4.54	3.22	4.64	3.39	4.49	3.30	4.81	3.50
5	4.57	3.45	4.69	3.44	4.70	3.49	4.54	3.30	4.93	3.04
6	4.63	3.28	4.39	3.33	4.73	3.48	4.60	3.30	4.55	3.36

Table 7.4: Plastic deformity (N/mm^2) in PMMA specimen following immersion

	No immersion	Oil	Soda	Milk	Caffeine
1	3259.700	2893.910	1654.210	2663.360	1914.350
2	3013.880	2649.280	1898.610	2753.400	2430.580
3	2892.070	1663.010	1984.010	2622.810	2264.990
4	2041.960	2621.890	2801.800	2633.420	2637.370
5	2119.110	2686.650	1509.200	2702.570	2171.250
6	2669.630	2495.140	2373.480	1957.330	2339.870

Table 7.5: Ultimate tensile stress (N) in PMMA specimen following immersion

	No immersion	Oil	Soda	Milk	Caffeine
1	843.967	693.963	869.560	670.226	641.670
2	511.284	732.635	670.513	625.525	764.281
3	679.747	672.805	808.001	572.414	722.017
4	836.550	720.415	452.395	752.948	618.099
5	705.493	719.595	619.157	786.991	441.961
6	810.420	423.203	710.630	304.133	649.544

Table 7.6: Plastic deformity (N/mm^2) in PLA specimen following immersion

	No immersion	Oil	Soda	Milk	Caffeine
1	1137.190	1364.530	1383.120	1454.770	1360.320
2	1389.230	1155.410	1395.740	1363.870	1273.190
3	1253.390	1407.710	1284.340	1456.420	1175.680
4	1313.440	1349.010	1415.230	1170.990	987.078
5	1171.770	1250.220	1353.750	1243.060	1475.810
6	1077.910	1240.870	1439.110	1226.660	1397.890

Table 7.7: Ultimate tensile stress (N) outcomes in PLA specimen following immersion

	No immersion	Oil	Soda	Milk	Caffeine
1	357.199	393.534	390.848	340.894	406.202
2	382.977	336.603	345.939	368.493	377.865
3	334.336	370.118	329.434	348.978	354.417
4	358.696	383.345	342.175	358.149	312.796
5	324.116	345.163	342.499	355.504	431.388
6	324.218	318.394	344.779	364.463	413.106

Table 7.8: Plastic deformity (N/mm^2) in PETG specimen following immersion

	No immersion	Oil	Soda	Milk	Caffeine
1	1273.230	1965.810	1796.920	683.568	708.084
2	1991.020	1135.980	1135.420	1133.050	1000.100
3	1121.650	1234.550	765.169	956.414	860.449
4	589.661	1200.070	1272.810	1149.030	835.762
5	1152.610	1036.980	1178.780	1204.700	1142.100
6	1090.110	1280.480	1051.450	1257.760	1004.470

Table 7.9: Ultimate tensile stress (N) outcomes in PETG specimen following immersion

	No immersion	Oil	Soda	Milk	Caffeine
1	392.297	550.954	520.248	167.259	121.810
2	502.501	314.458	333.481	255.117	229.572
3	209.773	296.294	179.510	204.061	258.188
4	97.507	310.408	352.872	295.836	183.608
5	263.602	318.867	273.008	314.884	491.486
6	230.351	334.285	213.153	345.545	266.301

8. Flexural strength data

Table 8.1: Flexural strength linear measurements (in mm) for PMMA (n=30)

	No immersion		Cooking oil		Soda		Milk		Caffeine	
	breadth	*Height*	*breadth*	*Height*	*breadth*	*Height*	*breadth*	*Height*	*breadth*	*Height*
1	9.86	3.18	10.01	3.80	10.04	3.41	9.91	3.94	9.94	4.06
2	9.98	3.35	9.97	3.28	10.12	3.67	9.86	3.46	9.91	4.14
3	10.05	3.48	10.19	3.42	10.05	3.40	9.82	3.54	9.87	3.91
4	10.17	3.37	9.96	3.29	10.00	3.44	9.98	3.61	10.00	3.36
5	10.02	3.42	9.89	3.09	9.96	3.50	9.88	3.20	9.88	3.91
6	9.82	3.16	9.99	3.31	9.88	3.46	10.11	3.45	10.28	3.63

Table 8.2: Flexural strength linear measurements (in mm) for PLA (n=30)

	No immersion		Cooking oil		Soda		Milk		Caffeine	
	breadth	*Height*	*breadth*	*Height*	*breadth*	*Height*	*breadth*	*Height*	*breadth*	*Height*
1	9.98	2.90	10.19	3.15	9.93	3.01	9.89	2.95	9.81	3.17
2	10.01	3.13	10,02	3.15	9.97	3.01	9.98	2.99	9.88	3.31
3	9.92	3.11	9.99	3.13	10.05	3.03	9.87	3.13	9.90	3.27
4	9.96	2.96	10.05	3.07	10.01	3.12	9.92	3.05	9.86	3.07
5	9.96	3.11	10.07	3.10	9.95	2.98	9.93	2.94	9.80	3.10
6	10.02	3.12	10.03	3.01	9.93	3.02	9.92	3.13	9.85	3.21

Table 8.3: Flexural strength linear measurements (in mm) for PETG (n=30)

	No immersion		Cooking oil		Soda		Milk		Caffeine	
	breadth	*Height*	*breadth*	*Height*	*breadth*	*Height*	*breadth*	*Height*	*breadth*	*Height*
1	9.81	3.29	9.40	3.32	9.91	3.04	9.89	2.95	9.88	3.29
2	9.85	3.18	9.86	3.26	9.85	3.13	9.98	2.99	9.86	3.02
3	9.81	3.07	9.87	2.98	8.84	3.25	9.87	3.13	9.89	3.26
4	9.78	3.20	9.94	3.11	9.67	3.09	9.92	3.05	9.89	3.16

5	9.84	3.18	9.76	3.31	9.87	3.16	9.93	2.94	9.83	3.04
6	9.87	3.21	9.85	3.08	9.90	3.18	9.92	3.13	9.85	3.21

Table 8.4: Flexural deformity (N/mm^2) in PMMA specimen following immersion

	**No immersion**	**Oil**	**Soda**	**Milk**	**Caffeine**
1	382.340	1420.540	1496.650	971.533	1480.510
2	953.094	1612.700	1469.610	1496.120	1276.800
3	379.479	1301.430	1794.190	1529.390	980.813
4	101.750	2224.860	1660.980	1550.320	1742.160
5	1073.710	1248.010	2325.390	2113.830	1021.980
6	718.255	1794.790	2033.840	1943.020	2215.430

Table 8.5: Break force (N) in PMMA specimen following immersion

	**No immersion**	**Oil**	**Soda**	**Milk**	**Caffeine**
1	196.997	187.623	195.999	185.410	275.237
2	156.864	147.597	272.694	215.772	242.933
3	215.314	200.930	127.227	212.520	194.766
4	185.957	206.617	214.507	237.195	183.916
5	220.372	201.909	214.402	179.742	193.761
6	153.818	238.921	211.859	202.382	247.154

Table 8.6: Flexural deformity (N/mm^2) in PLA specimen following immersion

	**No immersion**	**Oil**	**Soda**	**Milk**	**Caffeine**
1	1458.830	965.410	1534.730	1536.320	1376.110
2	1327.130	1719.980	1609.010	1483.710	1345.280
3	1350.480	1156.950	1519.660	1374.860	1448.470
4	1475.270	1452.820	1806.850	1520.060	1266.190
5	1451.330	1535.240	1374.450	1398.730	1322.470
6	1400.030	1573.740	1452.570	1430.130	1368.240

Table 8.7: Break force (N) in PLA specimen following immersion

	No immersion	Oil	Soda	Milk	Caffeine
1	76.297	87.061	70.604	54.356	70.864
2	61.693	68.324	64.659	48.863	68.356
3	80.579	70.693	67.892	67.650	74.555
4	72.174	62.939	69.017	72.927	50.522
5	81.135	83.176	64.020	56.508	68.925
6	84.368	67.558	52.593	46.895	66.020

Table 8.8: Flexural deformity (N/mm^2) in PETG specimen following immersion

	No immersion	Oil	Soda	Milk	Caffeine
1	1163.270	1790.630	1539.210	1759.410	1633.750
2	1275.930	1215.610	1729.200	1574.590	1463.150
3	840.843	1590.900	1633.480	1732.860	1678.340
4	1526.890	1552.430	1526.790	1716.410	1670.450
5	1594.760	1688.930	1525.790	1417.620	1784.850
6	1573.740	1701.690	1429.550	1734.090	1678.300

Table 8.9: Break force (N) in PETG specimen following immersion

	No immersion	Oil	Soda	Milk	Caffeine
1	94.728	110.238	81.711	114.981	94.964
2	112.880	101.147	115.906	96.931	83.024
3	104.434	76.771	113.112	138.636	105.483
4	92.478	79.896	86.527	114.339	110.785
5	114.724	115.875	92.513	103.102	105.047
6	114.918	87.442	94.601	87.423	111.405
